# Common driver mutations and programmed death-ligand 1 expression in advanced non-small cell lung cancer in smokers and never smokers

**DOI:** 10.1186/s12885-023-11156-y

**Published:** 2023-07-14

**Authors:** Chong Kin LIAM, Chian Yih YEW, Yong Kek PANG, Chee Kuan WONG, Mau Ern POH, Jiunn Liang TAN, Chun Ian SOO, Thian Chee LOH, Ka Kiat CHIN, Vijayan MUNUSAMY, Yong Sheng LIAM, Nur Husna IBRAHIM

**Affiliations:** 1 Department of Medicine, Faculty of Medicine, University of Malaya, 50603 Kuala Lumpur, Malaysia; 2 Department of Medicine, University of Malaya Medical Centre, 59100 Kuala Lumpur, Malaysia; 3 Clinical Investigation Centre, University of Malaya Medical Centre, 59100 Kuala Lumpur, Malaysia

**Keywords:** *EGFR* mutations, NSCLC, PD-L1, Smoking status

## Abstract

**Background:**

In non-small cell lung cancer (NSCLC), there may be a relationship between programmed death-ligand 1 (PD-L1) expression, driver mutations and cigarette smoking.

**Methods:**

In this single-center retrospective study, the relationship between common driver mutations (*EGFR* mutation and *ALK* rearrangement) and PD-L1 expression in advanced NSCLC according to the patients’ smoking history was examined. Light, moderate and heavy smokers had smoked < 20, 20–39, and ≥ 40 pack-years, respectively. The level of PD-L1 expression, assessed using Ventana SP263 monoclonal antibody assay, was defined by the tumor proportion score (TPS) as high expression (TPS ≥ 50%), low expression (TPS 1%—49%) and no expression (TPS < 1%).

**Results:**

101 (52.9%) of 191 advanced NSCLC patients were never smokers. *EGFR* mutations were more common in never smokers (64.4%) than in smokers (17.8%) with advanced NSCLC (*P* < 0.0001). A higher proportion of smokers (26.7%) had high PD-L1 expression compared to never smokers (13.9%) (*P* = 0.042). There was a trend for a higher proportion of male NSCLC patients [28 of 115 (24.3%)] than female patients [10 of 76 (13.2%)] to have high PD-L1 expression (*P* = 0.087]. High PD-L1 expression was seen in 32 of 110 (29.1%) patients with *EGFR* wild-type NSCLC but only in 6 of 81 (7.4%) patients with *EGFR*-mutant tumors (*P* < 0.0001). Among the 90 smokers with NSCLC, a higher proportion of heavy smokers (35.8%) than non-heavy smokers (13.5%) had high PD-L1 expression (*P* = 0.034). In patients with adenocarcinoma, high PD-L1 expression was seen in 25 of 77 (32.5%) patients with *EGFR* wild-type tumors but only in 4 of 70 (5.7%) patients with *EGFR*-mutant tumors (*P* < 0.0001). Among patients with adenocarcinoma, a significantly higher proportion of ever smokers (29.3%) than never smokers (13.5%) had high PD-L1 expression (*P* = 0.032). Among smokers with adenocarcinoma, a significantly higher proportion of heavy smokers (44.1%) than non-heavy smokers (8.3%) had high PD-L1 expression (*P* = 0.004). On multivariate analysis, after adjusting for gender and smoking status, heavy smoking and *EGFR* wild-type tumors remained significantly associated with high PD-L1 expression in NSCLCs and also in adenocarcinoma.

**Conclusions:**

Heavy smoking and *EGFR* wild-type tumors were significantly associated with high PD-L1 expression in NSCLCs and also in adenocarcinoma.

## Key messages

Significant findings of the study

In advanced NSCLC, particularly in advanced adenocarcinoma, high PD-L1 expression is significantly more common in smokers than in never smokers, in *EGFR* wild-type than *EGFR*-mutant tumors in both males and females and in heavy smokers among smokers. On multivariate analysis, NSCLCs including adenocarcinoma in patients who are heavy smokers and which are *EGFR* wild-type are significantly associated with high PD-L1 expression.

What this study adds

In a region where a high proportion of NSCLC patients are never smokers and *EGFR* mutation is highly prevalent, high PD-L1 expression in NSCLC and adenocarcinoma in particular is significantly associated with heavy smoking and *EGFR* wild-type tumors.

## Introduction

Lung cancer is the second most common malignancy and the leading cause of cancer-related mortality worldwide [1]. Non-small cell lung cancer (NSCLC) accounts for 85% or more of lung cancer with adenocarcinoma being the most prevalent NSCLC subtype. The most common actionable driver mutations in NSCLC are epidermal growth factor receptor (*EGFR*) mutation and anaplastic lymphoma kinase (*ALK*) rearrangement [2]. Activating mutations in the *EGFR* gene occur in 10–20% of Caucasian but in up to 60% of Asian NSCLC patients [3]. *EGFR* mutations have been found to be present in about 40% of advanced lung adenocarcinoma and 12% of squamous cell carcinoma in Malaysian patients [4, 5]. *ALK* rearrangement occurs in 5% to 6% of NSCLC cases and is more common in younger patients who are never or light smokers with adenocarcinoma histology irrespective of gender [6, 7]. About 13% of *EGFR* wild-type adenocarcinoma in Malaysian patients harbour *ALK* alteration [8].

The more common actionable driver alterations are until very recently individually identified in biopsy specimens using sequential single-gene testing because these alterations are generally mutually exclusive [9, 10]. The selection of treatment regimens for NSCLC is now dependent on not only the histological subtypes, but also the status of driver mutations and the programmed death-ligand 1 (PD-L1) expression of the lung cancer. Accurate biomarker evaluation plays an important role in the personalized treatment of NSCLC. The PD-L1 expression of NSCLC and three driver alterations including *EGFR* mutation, *ALK* rearrangement and *ROS1* rearrangement are recommended as the necessary biomarkers to be tested by our national consensus statement [11].

Previous studies conducted in the West [12–15] and in Asia [16] have shown an inverse relationship between PD-L1 expression and *EGFR* mutations in NSCLC and a correlation of high PD-L1 expression with tobacco smoking [15, 17]**. **There are no local Malaysian studies on the smoking status of patients and the degree of PD-L1 expression in NSCLC. This study aimed to determine the relationship between the presence of common driver alterations and PD-L1 expression in NSCLC with smoking in a Malaysian population which, similar to other Asian populations, is known to have a high prevalence of *EGFR* mutation and a lower proportion of smokers compared to the West [3, 4, 18].

We conducted a retrospective study to examine the relationship between common driver gene alterations (*EGFR* mutation, *ALK* rearrangement and *ROS1* rearrangement) and PD-L1 expression in NSCLC in smokers and never smokers.

## Methods

This is a a single-center retrospective study of patients with newly diagnosed locally advanced or metastatic stage NSCLC treated at the Division of Respiratory Medicine, Department of Medicine, University of Malaya Medical Centre (UMMC), Kuala Lumpur during a 2-year period from 1 July 2019 to 30 June 2021.

Demographic characteristics of the patients including age, gender, intensity and duration of cigarette smoking; and clinical parameters including disease stage, histopathology, PD-L1 immunohistochemistry, and *EGFR* mutation, *ALK* and *ROS1* rearrangement results were obtained from the electronic medical records of the UMMC hospital information system. The clinical staging of the patient’s lung cancer according to the 8th edition of the American Joint Committee on Cancer (AJCC) tumor, node and metastasis (TNM) staging system for lung cancer [19] was based on clinical examination findings, contrast-enhanced computed tomography (CECT) scan of the thorax, abdomen and pelvis and/or positron emission tomography (PET)-CT scan, with or without brain CT scan or brain magnetic resonance imaging (MRI) when indicated by the need to exclude intracranial metastases depending on the presence of neurological symptoms and/or signs.

Testing for molecular biomarkers and PD-L1 expression was performed on biopsy specimens obtained from either the primary lung tumor or metastatic lesions. *EGFR* mutations were identified from formalin-fixed paraffin embedded (FFPE) histopathology biopsy specimens using the Roche Diagnostics cobas® *EGFR* Mutation Test allele-specific real-time polymerase chain reaction assay (cobas®, Roche Molecular System Inc, Branchburg, New Jersey, USA). The test kit was able to detect exon 19 deletion mutation and exon 21 L858R point mutation in the *EGFR* gene which are the two most commonly identified mutations, as well as rarer mutations which include exon 18 G719X mutation, exon 20 T790M mutation, exon 20 S7681 mutation, exon 20 insertion mutation, exon 21L861Q mutation, and exon 21 deletion mutation. Tumors in which any of these *EGFR* mutations were detected were considered *EGFR* mutation-positive.

For *ALK* rearrangement detection, fully automated immunohistochemical (IHC) staining using the Roche Diagnostics Ventana anti-ALK (D5F3) Rabbit Monoclonal Primary Antibody (Ventana IHC, Ventana, Tucson, Arizona, USA) was employed. This antibody test detects *ALK* protein in FFPE histopathology biopsy specimens by binding to the *ALK* protein, that can then be visualized using OptiView DAB IHC Detection Kit (manufactured in Tucson, Arizona, USA), followed by the OptiView Amplification Kit on a Benchmark IHC/ISH instrument (manufactured in Tucson, Arizona, USA). Detection of strong granular cytoplasmic staining was considered as a positive finding.

*ROS1* rearrangements at the 6q22 location of the *ROS1* gene were detected by fluorescence in situ hybridization (FISH) using the Vysis *ROS1* Break Apart FISH Probe Kit (Abbott Molecular Inc.,Abbot Park, Illinois, USA). 6q22 ROS1 Break Apart FISH (Abbott Molecular Inc.) utilizes the 6q22 ROS1 (Tel) SpectrumOrange Probe and 6q22 ROS1 (Cen) SpectrumGreen Probe for the detection of *ROS1* rearrangement in FFPE histopathology biopsy specimens. Hybridization of the ROS1 probes was viewed using a fluorescence microscope equipped with appropriate excitation and emission filters, allowing visualization of the orange and green fluorescent signals. A rearrangement-positive cell rate of ≥ 15% was interpreted as *ROS1*-positive.

Testing for *ALK* was only carried out in biopsy specimens which were tested negative for *EGFR* mutation and testing for *ROS1* rearrangement was only carried out when both *EGFR* and *ALK* alterations were not detected in a 3-step testing protocol since these molecular alterations are mutually exclusive [11].

For PD-L1 detection and quantification of the degree of expression, Roche Diagnostics Ventana PD-L1 (SP263) Assay (Tucson, Arizona, USA) was used which is intended for the qualitative detection of the PD-L1 protein in NSCLC. It utilizes a rabbit monoclonal antibody that binds to PD-L1 in FFPE tissue sections. The specific antibody is visualized using OptiView DAB IHC Detection Kit on a Benchmark IHC/ISH instrument. The degree of PD-L1 expression is defined by the tumor proportion score (TPS) which is the percentage of tumor cells showing partial or complete membrane staining for PD-L1. The level of PD-L1 expression defined by TPS was as follows: high expression (TPS ≥ 50%), low expression (TPS 1%—49%) and no expression (TPS < 1%).

Detailed smoking histories of the patients were prospectively collected. A never smoker was someone who had never smoked or smoked less than 100 cigarettes in a lifetime. Light, moderate, and heavy smokers were patients who had smoked less than 20, 20 to 39, and 40 or more pack-years, respectively [20]. Pack-year is defined as the average number of packs of cigarettes smoked per day (with one pack = 20 cigarettes) multiplied by the number of years of smoking. Former smokers were defined as smoking previously of at least 100 cigarettes in his/her lifetime but who were no longer smoking for at least one year.

The study was approved by the hospital ethics committee. Patient confidentiality was maintained by anonymizing patient data to remove any identifying information. Written informed consent by the patients was not required for this study in accordance with the national legislation and the institutional requirements.

### Statistical analysis

All statistical analyses were performed using the Statistical Package for Social Science (SPSS) for Windows version 26.0 (SPSS Corp., Chicago, IL, USA). In the analysis of the demographic and clinical data of the patients, results for continuous variables were expressed as median and range. Results for categorical variables were expressed as absolute frequencies and percentages. Differences between groups were tested for significance with the Pearson Chi-square test or Fisher’s exact test whichever was appropriate for categorical variables. A two-sided p-value of less than 0.05 was considered statistically significant. Multivariate analysis using logistic regression was performed to determine the association between clinical parameters and high PD-L1 expression.

## Results

### Demographic and clinical characteristics of the patients

The demographic data, clinical characteristics, *EGFR* mutation and *ALK* status, and PD-L1 expression level of a total of 191 study patients are shown in Table 1.

**Table 1:** Demographic and clinical characteristics, *EGFR* and *ALK* alteration status and PD-L1 expression of NSCLC of 191 patients

Characteristic	No. of patients (%)
**Age** (median) (interquartile range) 67 (59 – 73) years	191
**Gender**	
**Male**	115 (60.2%)
**Female**	76 (39.8%)
**Smoking status**	
**Never smoker**	101 (52.9%)
**Ever smoker**	90 (47.1%)
**Light smoker**	16/90 (17.8%)
**Moderate smoker**	21/90 (23.3%)
**Heavy smoker**	53/90 (58.9%)
**NSCLC histological subtype**	
**Adenocarcinoma**	147 (77.0%)
**Squamous cell carcinoma**	34 (17.8%)
**Adenosquamous carcinoma**	9 (4.7%)
**Mucoepidermoid carcinoma**	1 (0.5%)
**AJCC (8th edition) stage at diagnosis**	
**Locally advanced disease (stage IIIA, IIIB, IIIC)**	22 (11.5%)
**Metastatic disease (IVA, IVB)**	169 (88.5%)
***EGFR* mutation**	
**Positive**	81 (42.4%)
**Exon 18 G719X mutation**	3/81 (3.7%)
**Exon 19 deletion mutation**	28/81 (34.6%)
**Exon 20 insertion mutations**	5/81 (6.2%)
**Exon 21 L858R mutation**	4/81 (54.3%)
**Exon 18 G719X and exon 21 L861Q mutations**	1/81 (1.2%)
**Negative**	110 (57.6%)
***ALK***	
**Positive**	8 (4.2%)
**Negative**	183 (95.8%)
**Degree of PD-L1 expression**	
**TPS ≥ 50%** (high expression)	38 (19.9%)
**TPS 1–49%** (low expression)	81 (42.4%)
**TPS < 1%** (no expression)	72 (37.7%)

*TPS* = Tumor proportion score

All the patients were living in the most populated and urbanised area in Malaysia, the Klang Valley with a population of 8 million in which the capital city of Kuala Lumpur is situated. The overall quality of air in the Klang Valley as determined by the Air Pollution Index (API) which is mainly based on five major pollutants (PM_10_, SO_2_, NO_2_, CO, and O_3_) in the ambient air was considered moderate [21]. The age of the study patients ranged from 32 to 89 years with a median of 67 (interquartile range, 59 – 73) years and 71.2% of the patients were aged 60 years or above. The male to female ratio of the patients was 3:2. Slightly more than half of the patients (52.9%) were never smokers. Of the 90 patients who were ever smokers, 16 (17.8%), 21 (23.3%) and 53 (58.9%) were light, moderate and heavy smokers, respectively. The majority, 147 (77.0%) of the NSCLC cases were adenocarcinoma, while 34 (17.8%) were squamous cell carcinoma (SCC) and 9 (4.7%) were adenosquamous carcinoma. An overwhelming majority of the patients (88.5%) had stage IV disease at diagnosis.

*EGFR* mutations were detected in 81 patients. Of these, 72 were common activating *EGFR* mutations (exon 19 deletion and exon 21 L858R mutations) as shown in Table 1. Three patients had exon 18 G719X mutation, five patients had *EGFR* exon 20 insertion mutations and one patient had a combination of exon 18 G719X and exon 21 L861Q mutations.

*ALK* was detected in only 8 (4.2%) of the 191 patients. Since *ALK* and *EGFR* mutations are mutually exclusive, *ALK* was only tested in tumors which were *EGFR* wild-type (i.e., *EGFR* mutation-negative) in our center. The *ALK*-positive rate among our 110 *EGFR* wild-type NSCLC patients was 7.3%. Only one patient was tested positive for *ROS1* rearrangement.

The tumors of 37.7% of the patients did not express PD-L1 while that of 42.4% and 19.9% had low and high PD-L1 expression, respectively.

Table 2 shows the gender of the patients, the presence of common oncogenic driver alterations (i.e., *EGFR* mutation or *ALK*) and the level of PD-L1 expression according to the patients’ smoking status. A significantly higher proportion of male patients (73.9%) were smokers while 93.4% of female patients were never smokers. Only five (6.6%) female patients were smokers. *EGFR* mutations were significantly more common in never smokers [65 (64.4%) of 101 patients] than in smokers [16 (17.8%) of 90 patients] (*P* < 0.0001). *ALK* positivity was more common among never smokers but the difference from smokers was not significant because of the small number of *ALK*-positive patients. The *ROS1*-positive patient was a never smoker and her tumor did not express PD-L1. A significantly higher proportion of smokers had high PD-L1 expression (TPS ≥ 50%) [24 (26.7%) of 90] compared to never smokers [14 (13.9%) of 101] (*P* = 0.042).

**Table 2:** *EGFR* mutation, *ALK* and PD-L1 expression in NSCLC according to the patients’ smoking status (*N* = 191)

**Characteristic**	**No. of patients (%)** (Total = 191)			***P* value**
**Smoking status**	**Total**	**Ever smoker**	**Never smoker**	
		*n* = 90 (47.1%)	*n* = 101 (52.9%)	
**Gender**				
**Male**	115 (60.2%)	85 (73.9%)	30 (26.1%)	< 0.0001
**Female**	76 (39.8%)	5 (6.6%)	71 (93.4%)	
***EGFR* mutation**				
**Positive**	81 (42.4%)	16 (17.8%)	65 (64.4%)	< 0.0001
**Negative**	110 (57.6%)	74 (82.2%)	36 (35.6%)	
***ALK***				
**Positive**	8 (4.2%)	2 (2.2%)	6 (5.9%)	0.285
**Negative**	183 (95.8%)	88 (97.8%)	95 (94.1%)	
**PD-L1 expression**				
**TPS ≥ 50%** (High expression)	38 (19.9%)	24 (26.7%)	14 (13.9%)	0.042*
**TPS 1% to 49%** (Low expression)	81 (42.4%)	35 (38.9%)	46 (45.5%)	
**TPS < 1%** (No expression)	72 (37.7%)	31 (34.4%)	41 (40.6%)	

*TPS* = Tumor proportion score

^*^Comparison between high PD-L1 expression (TPS ≥ 50%) versus no (TPS < 1%) and low PD-L1 expression (TPS 1%-49%) in ever smoker versus never smoker

The proportions of male and female patients with *EGFR* mutation and *ALK* are shown in Table 3. A significantly higher percentage of female patients were *EGFR* mutation-positive. *ALK* positivity was more common among female patients but the difference from male patients was not significant because of the small number of patients with *ALK*.

**Table 3:** *EGFR* mutation and *ALK* in NSCLC according to the patients’ gender

	**No. of patients (%)** (Total = 191)			***P* value**
**Characteristics**	**Total**	**Male**	**Female**	
*EGFR* mutation-positive	81 (42.4%)	31 (27.0%)	50 (65.8%)	< 0.0001
*ALK*-positive	8 (4.2%)	3 (2.6%)	5 (6.6%)	0.269

Table 4 shows *EGFR* mutation and *ALK* status according to the histological subtype of NSCLC. A significantly higher proportion of patients with adenocarcinoma [70 of 147 (47.6%)] were *EGFR* mutation-positive compared to patients with SCC [7 of 34 (20.6%)] (*P* = 0.007). The frequency of *EGFR* mutation in adenosquamous carcinoma (44.4%) is similar to that in adenocarcinoma. *ALK* was only detected in patients with adenocarcinoma.

**Table 4:** *EGFR* mutation and *ALK* according to histological subtype of NSCLC

	**No. of patients (%)**		
**Histological subtype**	**All patients**	***EGFR* mutation-positive**	***ALK*-positive**
Adenocarcinoma	147 (77.0%)	70 (47.6%)	8 (5.4%)
Squamous cell carcinoma	34 (17.8%)	7 (20.6%)	0
Adenosquamous carcinoma	9 (4.7%)	4 (44.4%)	0
Mucoepidermoid carcinoma	1 (0.5%)	0	0

Table 5 shows the level of PD-L1 expression according to the patients’ gender, common driver mutation status, smoking status, the intensity of cigarette smoking among the ever smokers, and histological subtype. There was a trend for a higher proportion of male patients to have high PD-L1 expression but the difference from female patients was not statistically significant [28 of 115 male patients (24.3%) versus 10 of 76 female patients (13.2%), *P* = 0.087]. 48 of 81 (59.3%) tumors with *EGFR* mutations expressed PD-L1 while 71 of 110 (64.5%) tumors without *EGFR* mutations expressed PD-L1 (*P* = 0.553). High PD-L1 expression was seen in 32 of 110 patients (29.1%) with *EGFR* wild-type tumors but only in 6 of 81 (7.4%) patients with *EGFR*-mutant tumors (*P* < 0.0001) (Table 5). Table 6 shows the tumor PD-L1 expression according to the *EGFR* mutation subtype. The majority of tumors harboring uncommon *EGFR* mutations (exon 18 G719X and exon 20 insertion and exon 21 L861Q mutations) did not express PD-L1 or had low PD-L1 expression. Seven of 8 tumors with *ALK* expressed no or low PD-L1 expression (*P* = 1.000).

**Table 5:** PD-L1 expression in NSCLC according to gender, common driver mutation status, smoking status and histological subtype (*N* = 191)

Characteristic	No. of patients (%)			*P* value*
**PD-L1 expression**	**TPS < 1%** (No expression)	**TPS 1% to 49%** (Low expression)	**TPS ≥ 50%** (High expression)	
**Gender**				
Male (*n* = 115)	40 (34.8%)	47 (40.9%)	28 (24.3%)	0.087
Female (*n* = 76)	32 (42.1%)	34 (44.7%)	10 (13.2%)	
***EGFR* mutation**				
Present	33 (40.7%)	42 (51.9%)	6 (7.4%)	< 0.0001
Absent	39 (35.5%)	39 (35.5%)	32 (29.1%)	
***ALK***				
Present (*n* = 8)	1 (12.5%)	6 (75.0%)	1 (12.5%)	1.000
Absent (*n* = 183)	71 (38.8%)	75 (41.0%)	37 (20.2%)	
**Smoking status**				
Never smoker (*n* = 101)	41 (40.6%)	46 (45.5%)	14 (13.9%)	0.042
Ever smoker (*n* = 90)	31 (34.4%)	35 (38.9%)	24 (26.7%)	
**Pack-years of smoking among ever smokers** (*n* = 90)				
< 20 (Light smoker *n* = 16)	8 (50.0%)	7 (43.8%)	1 (6.3%)	0.034
20–39 (Moderate smoker *n* = 21)	6 (28.6%)	11 (52.4%)	4 (19.0%)	
**≥ **40 (Heavy smoker *n* = 53)	17 (32.1%)	17 (32.1%)	19 (35.8%)	
**Ever smoker** (*n* = 90)				
Current smoker (*n* = 53)	20 (37.7%)	17 (32.1%)	16 (30.2%)	0.508
Former smoker (*n* = 37)	11 (29.7%)	18 (48.6%)	8 (21.6%)	
**Histological subtype of NSCLC**				
Squamous cell (*n* = 34)	12 (35.3%)	15 (44.1%)	7 (20.6%)	1.000
Adenocarcinoma (*n* = 147)	57 (38.8%)	61 (41.5%)	29 (19.7%)	

*TPS* = Tumor proportion score

^*^In each group, comparison is between high PD-L1 expression (TPS ≥ 50%) versus no (TPS < 1%) and low PD-L1 expression (TPS 1%-49%)

**Table 6:** PD-L1 expression in NSCLC according to *EGFR* mutation subtype (*n* = 81)

	**PD-L1 expression [No. of patients (%)]**		
	**TPS < 1%** (No expression)	**TPS 1% to 49%** (Low expression)	**TPS ≥ 50%** (High expression)
***EGFR* mutation subtype**			
Exon 18 G719X mutation (*n* = 3)	0	2 (66.7%)	1 (33.3%)
Exon 19 deletion mutation (*n* = 28)	13 (46.4%)	15 (53.6)	0
Exon 20 insertion mutations (*n* = 5)	3 (60.0%)	1 (20.0%)	1 (20.0%)
Exon 21 L858R mutation (*n* = 44)	16 (36.4%)	24 (54.5%)	4 (9.1%)
Exon 18 G719X and exon 21 and L861Q mutations (*n* = 1)	1 (100.0%)	0	0

The tumors of 59 of 90 (65.6%) of smokers expressed PD-L1 while that of 60 of 101 (59.4%) never smokers expressed PD-L1 (*P* = 0.468) (Table 5). Among the 90 smokers, a significantly higher proportion of heavy smokers [19 (35.8%) of 53] than non-heavy smokers [5 (13.5%) of 37] had high PD-L1 expression [OR, 1.5 (95% CI, 1.1 – 2.1), *P* = 0.034] (Table 5). Among ever smokers, the level of PD-L1 expression was not affected by whether the smoker was a current smoker or a former smoker (*P* = 0.508) although there was a significantly higher proportion of heavy smokers [40 of 53 (75.5%) among current smokers compared to [13 of 37 (24.5%)] former smokers (*P* < 0.0001). There was no association between PD-L1 expression and the histological subtype of NSCLC [22 of 34 patients (64.7%) with SCC compared to 90 of 147 patients (61.2%) with adenocarcinoma had PD-L1 expression (*P* = 0.857)]. There was also no association between the level of PD-L1 expression and the histological subtype of NSCLC [7 of 34 patients (20.6%) with SCC compared to 29 of 147 patients (19.7%) with adenocarcinoma had high PD-L1 expression (*P* = 1.000)]. Among ever smokers with adenocarcinoma and ever smokers with SCC, the proportions of current smokers [37 of 58 (63.6%)] and [14 of 27 (51.9%)], respectively were not significantly different (*P* = 0.419). Among ever smokers with adenocarcinoma and those with SCC, the proportions of heavy smokers [34 of 58 (58.6%)] and [17 of 27 (63.0%), respectively were not significantly different (*P* = 0.887).

**Fig. 1:**
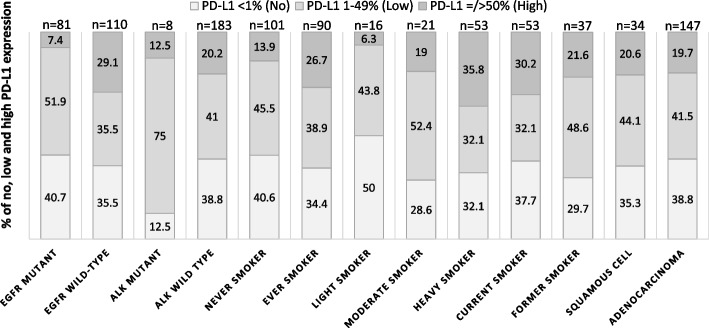
PD-L1 expression according to driver mutation status, smoking status and histological subtype

The distribution of PD-L1 expression according to the *EGF*R mutation and *ALK* status, smoking status, and histological subtype of NSCLC is shown in Fig. 1.

Table 7 shows the proportion of patients with *EGFR* mutation, *ALK* and the different levels of PD-L1 expression according to their gender and smoking status as well as smoking intensity. A significantly higher proportion of male patients who were never smokers [17 of 30 never smokers (56.7%)] compared to ever smokers [14 of 85 ever smokers (16.5%)] had *EGFR* mutation (*P* < 0.0001). Among male smokers, a significantly higher proportion of light smokers [6 of 16 (37.5%)] had *EGFR* mutation compared to moderate and heavy smokers [8 of 69 (11.6%)] (*P* = 0.032). The number of patients with *ALK* was too small for a difference to be demonstrated according to the intensity of smoking. A higher proportion of male smokers [23 of 85 (27.1%)] compared to never smokers [5 of 30 (16.7%)] had high PD-1 expression (*P* = 0.326). Among male smokers, a significantly higher proportion of heavy smokers [18 of 48 (37.5%)] had high PD-L1 expression compared to light and moderate smokers [5 of 37 (13.5%)] (*P* = 0.015).

**Table 7:** *EGFR* mutation, *ALK* and PD-L1 expression in NSCLC according to the patients’ gender and smoking status

	**No. of patients (%)**									
							**PD-L1 expression**			
	***EGFR* mutation-positive**	***EGFR* mutation-negative**	***P* value**	***ALK*-positive**	***ALK*-negative**	***P* value**	**TPS < 1%** (None)	**TPS 1% to 49%** (Low)	**TPS ≥ 50%** (High)	***P* value**
**Male** (*n* = 115)	31 (27.0%)	84 (73.0%)								
**Never smoker** (*n* = 30)	17 (56.7%)	13 (43.3%)	< 0.0001	1 (3.3%)	29 (96.7%)	1.000	11 (36.7%)	14 (46.7%)	5 (16.7%)	0.326**
**Ever smoker** (pack-years of smoking) (*n* = 75)	14 (16.5%)	71 (83.5%)		2 (2.4%)	83 (97.6%)		29 (34.1%)	33 (38.8%)	23 (27.1%)	
< 20 (Light smoker *n* = 16)	6 (37.5%)	10 (62.5%)	0.039^a^	1 (6.3%)	15 (93.8%)	0.254^a^	8 (50.0%)	7 (43.8%)	1 (6.3%)	0.015***
20–39 (Moderate smoker *n* = 21)	3 (14.3%)	18 (85.7%)		1 (4.8%)	20 (95.2%)		6 (28.6%)	11 (52.4%)	4 (19.0%)	
** ≥ **40 (Heavy smoker *n* = 48)	5 (10.4%)	43 (89.6%)		0	48 (100.0%)		15 (31.3%)	15 (31.3%)	18 (37.5%)	
**Female** (*n* = 76)	50 (65.8%)	26 (34.2%)		5 (7.0%)	66 (93.0%)		32 (42.1%)	34 (44.7%)	10 (13.2%)	
**Never smoker** (71)	48 (67.6%)	23 (32.4%)	0.331	5 (7.0%)	66 (93.0%)	1.000	30 (42.3%)	32 (45.1%)	9 (12.7%)	0.516**
**Ever smoker** (pack-years of smoking) (*n* = 5)	2 (40.0%)	3 (60.0%)		0	5 (100.0%)		2 (40.0%)	2 (40.0%)	1 (20.0%)	
< 20 (Light smoker *n* = 0)	0	0	-	0	0	-	0	0	0	-
20–39 (Moderate smoker *n* = 0)	0	0		0	0		0	0	0	
** ≥ **40 (Heavy smoker *n* = 5)	2 (40.0%)	3 (60.0%)		0	5 (100.0%)		2 (40.0%)	2 (40.0%)	1 (20.0%)	

*TPS* = Tumor proportion score

^a^Comparison between *EGFR* mutation-positivity or *ALK*-positivity in heavy smokers versus light and moderate smokers

^**^Comparison between high PD-L1 expression (TPS ≥ 50%) versus no (TPS < 1%) and low PD-L1 expression (TPS 1%-50%) in smokers versus never smokers

^***^Comparison between high PD-L1 expression (TPS ≥ 50%) versus no (TPS < 1%) and low PD-L1 expression (TPS 1%-50%) in heavy smokers versus light and moderate smokers

Although a higher proportion of female patients who were never smokers [48 of 71 (67.6%)] compared to ever smokers [2 of 5 (40.0%)] had *EGFR* mutation, the difference was not statistically significant (*P* = 0.331). Only 5 of our female patients were smokers, all of whom were heavy smokers. A higher proportion of female smokers [1 of 5 (20.0%)] compared to never smokers [9 of 71 (12.7%)] had high PD-1 expression (*P* = 0.516). The effect of smoking intensity on the presence or absence of *EGFR* mutation, *ALK* and the level of PD-1 expression could not be analysed in our female patients because all 5 were heavy smokers.

Table 8 shows the relationship between PD-L1 expression and *EGFR* mutation status among male and female NSCLC patients.

**Table 8:** PD-L1 expression in NSCLC according to the patients’ gender and *EGFR* mutation status

	**PD-L1 expression**			
	**TPS < 1%** (None)	**TPS 1% to 49%** (Low)	**TPS ≥ 50%** (High)	***P* value**
**Male** (*n* = 115)				
***EGFR* mutation-positive** (*n* = 31)	10 (32.3%)	18 (58.1%)	3 (9.7%)	0.028*
***EGFR* mutation-negative** (*n* = 84)	30 (35.7%)	29 (34.5%)	25 (29.8%)	
**Femal**e (*n* = 76)				
***EGFR* mutation-positive** (*n* = 50)	23 (46.0%)	24 (48.0%)	3 (6.0%)	0.026*
***EGFR* mutation-negative** (*n* = 26)	9 (34.6%)	10 (38.5%)	7 (26.9%)	

*TPS* = Tumor proportion score

^*^Comparison between high PD-L1 expression (TPS ≥ 50%) versus no (TPS < 1%) and low PD-L1 expression (TPS ≤ 50%) in *EGFR*-mutant versus *EGFR* wild-type NSCLC patients

Among male patients, 21 of 31 (67.7%) of *EGFR*-mutant tumors compared to 54 of 84 (64.3%) of *EGFR* wild-type tumors expressed PD-L1 (*P* = 0.901). In male patients, only 3 of 31 (9.7%) of *EGFR*-mutant tumors compared with 25 of 84 (29.8%) of *EGFR* wild-type tumors had high PD-L1 expression (*P* = 0.028). Among female patients, 27 of 50 (54.0%) of *EGFR*-mutant tumors compared to 17 of 26 (65.4%) of *EGFR* wild-type tumors expressed PD-L1 (*P* = 0.478). In female patients, only 3 of 50 (6.0%) of *EGFR*-mutant tumors compared with 7 of 26 (26.9%) of *EGFR* wild-type tumors had high PD-L1 expression (*P* = 0.026).

**Fig. 2:**
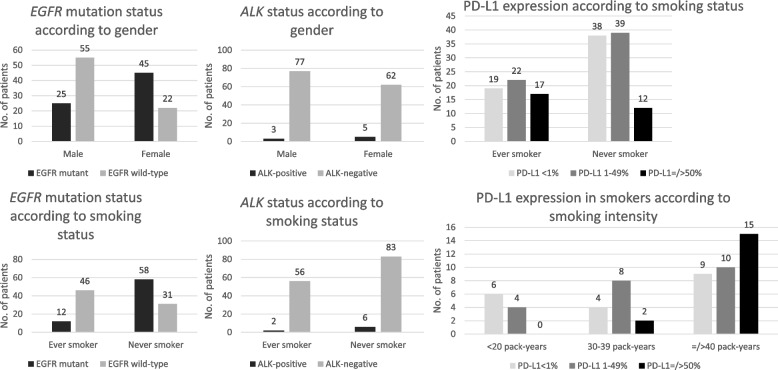
*EGFR* mutation, *ALK* and PD-L1 expression in adenocarcinoma according to the patients’ gender and smoking status

Table 9 shows *EGFR* mutation, *ALK* and PD-L1 expression in NSCLC according to the histologic subtype of NSCLC, the patients’ gender and smoking status. A significantly higher proportion of patients with adenocarcinoma [89 of 147 (60.5%)] were never smokers compared to those with SCC [7 of 34 (20.6%)] (*P* < 0.0001). Among the 147 patients with adenocarcinoma, a significantly higher proportion of never smokers [58 of 89 (65.2%)] had *EGFR* mutation compared to smokers [12 of 58 (20.7%)] (*P* < 0.0001). Among smokers with adenocarcinoma, the smoking intensity did not have an effect on *EGFR* mutation-positivity. Among the 147 patients with adenocarcinoma, the proportion of never smokers [6 of 89 (6.7%)] who had *ALK* was not different from that of smokers [2 of 58 (3.4%)] (*P* = 0.480). Among smokers with adenocarcinoma, only 2 patients who were non-heavy smokers were *ALK*-positive (*P* = 0.167). Among the 147 patients with adenocarcinoma, the tumors of 19 of 80 (23.8%) male patients expressed high PD-L1 compared to 10 of 67 (14.9%) female patients (*P* = 0.258). The tumors of 39 of 58 (67.2%) ever smokers expressed PD-L1 compared to 51 of 89 (57.3%) never smokers (*P* = 0.300). 48 of 81 (59.3%) tumors with *EGFR* mutations expressed PD-L1 while 71 of 110 (64.5%) tumors without *EGFR* mutations expressed PD-L1 (*P* = 0.553). High PD-L1 expression was seen in 25 of 77 patients (32.5%) with *EGFR* wild-type adenocarcinoma but only in 4 of 70 (5.7%) patients with *EGFR*-mutant tumors (*P* < 0.0001). A significantly higher proportion of ever smokers with adenocarcinoma [17 of 58 (29.3%)] had high PD-L1 expression compared to never smokers with adenocarcinoma [12 of 89 (13.5%)] (*P* = 0.032). Among smokers with adenocarcinoma, a significantly higher proportion of heavy smokers [15 of 34 (44.1%)] than non-heavy smokers [2 of 24 (8.3%)] had high PD-L1 expression (*P* = 0.004). Figure 2 shows the frequency of *EGFR* mutation, *ALK* and PD-L1 expression in adenocarcinoma according to the patients’ gender and smoking status.

**Table 9:** *EGFR* mutation, *ALK* and PD-L1 expression according to histologic subtype of NSCLC, patients’ gender and smoking status

	**No. of patients (%)**									
							**PD-L1 expression**			
	***EGFR* mutatio*n*-positive**	***EGFR* mutatio*n*-negative**	***P* value**	***ALK*-positive**	***ALK*-negative**	***P* value**	**TPS < 1%** (None)	**TPS 1% to 49%** (Low)	**TPS ≥ 50%** (High)	***P* value**
**Adenocarcinoma** (*n* = 147)	70 (47.6%)	77 (52.4%)		8 (5.4%)	139 (94.6%)		57 (38.8%)	61 (41.5%)	29 (19.7%)	
**Male** (*n* = 80)	25 (31.3%)	55 (68.8%)	< 0.0001	3 (3.8%)	77 (96.3%)	0.469	28 (35.0%)	33 (41.3%)	19 (23.8%)	0.258**
**Female** (*n*-67)	45 (67.2%)	22 (32.8%)		5 (7.5%)	62 (92.5%)		29 (43.3%)	28 (41.8%)	10 (14.9%)	
**Never smoker** (*n* = 89)	58 (65.2%)	31 (38.4%)	< 0.0001	6 (6.7%)	83 (93.3%)	0.480	38 (42.7%)	39 (43.8%)	12 (13.5%)	0.032**
**Ever smoker** (pack-years of smoking) (*n* = 58)	12 (20.7%)	46 (79.3%)		2 (3.4%)	56 (96.6%)		19 (32.8%)	22 (37.9%)	17 (29.3%)	
< 20 (Light smoker *n* = 10)	3 (30.0%)	7 (70.0%)	0.725^a^	1 (10.0%)	9 (90.0%)	0.167^a^	6 (60.0%)	4 (40.08%)	0	0.004***
20–39 (Moderate smoker *n* = 14)	3 (21.4%)	11 (78.6%)		1 (7.1%)	13 (92.9%)		4 (28.6%)	8 (57.1%)	2 (14.3%)	
** ≥ **40 (Heavy smoker *n* = 34)	6 (17.6%)	28 (82.4%)		0	34 (100.0%)		9 (26.5%)	10 (29.4%)	15 (44.1%)	
**Squamous cell carcinoma** (*n* = 34)	7 (20.6%)	27 (79.4%)		0	34 (100.0%)		12 (35.3%)	15 (44.1%)	7 (20.6%)	
**Male** (*n* = 28)	4 (14.3%)	24 (85.7%)	0.086	0	28 (100.0%)	-	10 (35.7%)	11(39.3%)	7 (25.0%)	0.306**
**Female** (*n* = 6)	3 (50.0%)	3 (50.0%)		0	6 (100.0%)		2 (33.3%)	4 (66.7%)	0	
**Never smoker** (7)	4 (57.1%)	3 (42.9%)	0.020	0	7 (100.0%)	-	1 (14.3%)	4 (57.1%)	2 (28.6%)	0.895**
**Ever smoker** (pack-years of smoking) (*n* = 27)	3 (11.1%)	24 (88.9%)		0	27 (100.0%)		11 (40.7%)	11 (40.7%)	5 (18.5%)	
< 20 (Light smoker *n* = 5)	2 (40.0%)	3 (60.0%)	0.535^a^	0	0	-	2 (40.0%)	2 (40.0%)	1 (20.0%)	0.326***
20–39 (Moderate smoker n = 5)	0	5 (100.0%)		0	0		1 (20.0%)	2 (40.0%)	2 (40.0%)	
** ≥ **40 (Heavy smoker *n* = 17)	1 (5.9%)	16 (94.1%)		0	0		8 (47.1%)	7 (41.2%)	2 (11.8%)	
**Adenosquamous carcinoma** (*n* = 9)	4 (44.4%)	5 (55.6%)		0	9 (100.0%)		2 (22.2%)	5 (55.6%)	2 (22.2%)	
**Male** (*n* = 6)	2 (33.3%)	4 (66.7%)	0.524	0	6 (100.0%)	-	1 (16.7%)	3 (50.0%)	2 (33.3%)	0.500**
**Female** (*n* = 3)	2 (66.7%)	1 (33.3%)		0	3 (100.0%)		1 (33.3%)	2 (67.7%)	0	
**Never smoker** (4)	3 (75.0%)	1 (25.0%)	0.206	0	4 (100.0%)		1 (25.0%)	3 (75.0%)	0	0.444**
**Ever smoker** (pack-years of smoking) (*n* = 5)	1 (20.0%)	4 (80.0%)		0	5 (100.0%)		1 (16.7%)	2 (33.3%)	3 (50.0%)	
< 20 (Light smoker *n* = 1)	1 (100.0%)	0		0	1 (100.0%)		0	1 (50.0%)	1 (50.0%)	0.100***
20–39 (Moderate smoker *n* = 2)	0	2 (100.0%)		0	2 (100.0%)		1 (50.0%)	1 (50.0%)	0	
** ≥ **40 (Heavy smoker *n* = 2)	0	3 (100.0%)		0	2 (100.0%)		0	0	2 (100.0%)	

*TPS* = Tumor proportion score

^a^Comparison between *EGFR* mutation-positivity or *ALK*-positivity in heavy smokers versus light and moderate smokers

^**^Comparison between high PD-L1 expression (TPS ≥ 50%) versus no (TPS < 1%) and low PD-L1 expression (TPS ≤ 50%) in male versus female and smokers versus never smokers

^***^Comparison between high PD-L1 expression (TPS ≥ 50%) versus no (TPS < 1%) and low PD-L1 expression (TPS ≤ 50%) in heavy smokers versus light and moderate smokers

Among the 34 patients with SCC, a significantly higher proportion of never smokers [4 of 7 (57.1%)] had *EGFR* mutation compared to smokers [3 of 27 (11.1%)] (*P* = 0.020). Among the smokers with SCC, the smoking intensity did not have an effect on *EGFR* mutation-positivity. Among the SCC patients, the tumors of 16 of 27 (59.3%) ever smokers expressed PD-L1 compared to 6 of 7 (85.7%) never smokers (*P* = 0.378). The proportion of ever smokers [5 of 27 (18.5%)] with SCC expressing high PD-L1 was not significantly different from that of never smokers [2 of 7 (28.6%)] (*P* = 0.615). Among smokers with SCC, 2 of 17 (11.8%) of heavy smokers and 3 of 10 (30.0%) non-heavy smokers had high PD-L1 expression (*P* = 0.326).

Table 10 shows the results of univariate and multivariate analyses of the effect of gender, smoking and *EGFR* mutation status on high PD-L1 expression of all NSCLCs and of adenocarcinoma. On multivariate analysis, after adjusting for gender and smoking status, heavy smoking and *EGFR* wild-type tumors remained significantly associated with high PD-L1 expression in NSCLCs and also in adenocarcinoma.

**Table 10:** Effect of gender, smoking and *EGFR* mutation status on high PD-L1 expression

**Parameter**	**All NSCLC (N = 191)**				**Adenocarcinoma (*n* = 147)**			
	**Univariate analysis**		**Multivariate analysis**		**Univariate analysis**		**Multivariate analysis**	
	**Likelihood estimate (95% CI)**	**P**	**Likelihood estimate (95% CI)**	**P**	**Likelihood estimate (95% CI)**	**P**	**Likelihood estimate (95% CI)**	**P**
Male vs. female	2.12 (0.96–4.68)	0.087	1.29 (0.44–3.78)	0.639	1.78 (0.76–4.14)	0.258	0.63 (0.16–2.51)	0.516
Ever smoker vs. never smoker	2.26 (1.09–4.70)	0.042	.0.48 (0.13–1.84)	0.286	2.66 (1.16–6.10)	0.032	0.41 (0.06–2.73)	0.354
Heavy smoker vs. never/non-heavy smoker	3.50 (1.67–7.35)	0.001	3.36 (1.10–10.23)	0.033	5.58 (2.32–13.44)	< 0.0001	8.58 (1.69–43.61)	0.010
*EGFR wild-type vs. EGFR*-mutant	5.13 (2.03–12.97)	< 0.0001	4.31 (1.56–11.96)	0.005	7.93 (2.60–24.22	< 0.0001	7.37 (2.22–24.48)	0.001

## Discussion

In this retrospective single center study, *EGFR* mutations were detected in 42.4% of the patients with NSCLC which corresponds to published data from Malaysia [4]. The higher prevalence of *EGFR* mutation in never smokers compared to ever smokers and in non-squamous NSCLC compared to squamous NSCLC is consistent with the established epidemiology of this molecular alteration in NSCLC [4, 5]. The frequency of the second commonest targetable driver alteration in our NSCLC patients, *ALK*, 4.2% in our patients is in keeping with the frequency of 5–6% of this molecular alteration in NSCLC described in the literature [6, 7, 22].

As this is a real-world study, the male to female ratio of 3:2 of our NSCLC patients is actually what is encountered in the clinic. A significantly higher proportion of our male patients who were never smokers (56.7%) compared to ever smokers (16.5%) had *EGFR* mutations. Among male smokers, a significantly higher proportion of light smokers (37.5%) compared to moderate and heavy smokers (11.6%) had *EGFR* mutations. Although a higher proportion of our female patients who were never smokers (67.6%) compared to ever smokers (40.0%) had *EGFR* mutations, the difference was not statistically significant because of the small number of female patients and only 5 of our female patients were smokers.

A significantly higher proportion of our patients with adenocarcinoma (60.5%) were never smokers compared to those with SCC (20.6%). A significantly higher proportion of patients with adenocarcinoma (47.6%) were *EGFR* mutation-positive compared to patients with SCC (20.6%). Among our 147 patients with adenocarcinoma, a significantly higher proportion of never smokers (65.2%) had *EGFR* mutation compared to smokers (20.7%). Similarly, among our 34 patients with SCC, a significantly higher proportion of never smokers (57.1%) had *EGFR* mutation compared to smokers (11.1%).

PD-L1 expression in tumor cells is associated with improved clinical outcomes of PD-1 pathway blockade in NSCLC patients [23, 24]. Several studies have also shown a relationship between high PD-L1 expression and a higher objective response rate and better survival in NSCLC patients treated with immune checkpoint inhibitors (ICIs) [25]. However, most clinical studies have excluded patients with *EGFR* mutations and *ALK*. Several United States of America Food and Drug Administration (FDA) approvals of ICIs were linked to specific PD-L1 thresholds [26].

The global multicenter EXPRESS study of 2368 NSCLC patients showed that 530 (22%) patients had PD-L1 TPS ≥ 50%, 1232 (52%) had PD-L1 TPS ≥ 1%, and 1136 (48%) had PD-L1 TPS < 1% [27]. In the EXPRESS study, percentages of patients with PD-L1 TPS ≥ 50% and TPS ≥ 1% are quite similar throughout the world being 22% and 52% in Europe; 22% and 53%, in Asia Pacific; 21% and 47% in the Americas; and 24% and 55%, respectively in the other countries. Our findings of 37.7% (TPS < 1%), 42.4% (TPS 1–49%), and 19.9% (TPS ≥ 50%) in our NSCLC patients are slightly different in terms of a higher percentage of patients with TPS ≥ 1% and a lower percentage of patients with TPS ≥ 50% which could be due to a lower percentage of smokers among our patients. However, the proportion of PD-L1 expression observed in our patient cohort is similar to other published studies, with a PD-L1 negative result in 39–41% of cases, low expression in 30–38% of cases and high expression in 21–30% of cases [28].

The results of PD-L1 from staining by using Ventana SP263 rabbit monoclonal antibody in our patients are considered interchangeable with results of PD-L1 staining by 2 other essays used in clinical practice, i.e., the (Dako 22C3 and Dako 28–8 monoclonal antibody clones) as shown by the PD-L1 IHC Blueprint Project [29].

Previous studies conducted in the West [12–15] and in Asia [16] have shown an inverse relationship between PD-L1 expression and *EGFR* mutations in NSCLC. Although the proportion of our patients with *EGFR*-mutant NSCLC expressing PD-L1 was not different from that of our patients with *EGFR* wild-type NSCLC, our study shows that high PD-L1 expression is significantly less frequently seen in *EGFR*-mutant NSCLC (7.4%) compared to *EGFR* wild-type tumors (29.1%). In both male and female NSCLC patients, significantly higher proportions of *EGFR* wild-type tumors had high PD-L1 expression compared to *EGFR*-mutant tumors. In the EXPRESS study, there is no difference in PD-L1 expression between tumors with driver mutations and those without [27]. Other studies have shown *EGFR-*mutant NSCLC patients have a lower chance of PD-L1 expression and PD-L1 expression or a strong PD-L1 expression has been reported to correlate with a worse outcome of EGFR-tyrosine kinase inhibitor (TKI) treatment [15, 30–33]. The results of a meta-analysis [of 47 studies with 11,444 patients – 23 investigated PD-L1 expression in NSCLC, 13 in adenocarcinoma, six in squamous cell carcinoma, 37 studies were conducted with Asian patients, and 10 studies were conducted with non-Asians patients; 23 studies included non-metastatic lung cancer patients, while 5 studies involved metastatic disease, and 17 studies involved both non-metastatic and metastatic diseases.] show that high PD-L1 expression is correlated with *EGFR* wild-type status [34]. The discrepancies among different studies might reflect the heterogeneous study population and variable definitions of PD-L1 expression. Our patients with exon 20 insertion mutations were considered *EGFR* mutation-positive since the demography of these patients is similar to those with common *EGFR* mutations [35].

Although the presence of *EGFR* mutation is inversely correlated with PD-L1 expression, the presence of *ALK* rearrangement has been shown by some studies to be associated with PD-L1 expression [12, 16, 36, 37]. In a study from Taiwan, the PD-L1 positive and strong positive rate among *ALK*-positive patients were 46.7% and 13.3%, respectively [32]. Our *ALK*-positive NSCLC cases were too few to demonstrate an association with PD-L1 expression. Although the number is small, 7 (87.5%) of our 8 *ALK*-rearranged NSCLC patients had PD-L1 expression compared to 48 (59.2%) of our 81 *EGFR*-mutant NSCLC patients. However, in the meta-analysis by Zhang et al., increased PD-L1 is not associated with *ALK* rearrangements [34].

A higher proportion of our NSCLC patients who were smokers had high PD-L1 expression compared to never smokers. A literature review by Norum and Nieder involving nine studies shows that NSCLC in smokers generally expresses higher PD-L1 [38]. High PD-L1 expression (≥ 50%) was correlated with current/ever smoking history in three of the nine studies [38]. A more recent study of a fairly large study population of 791 Caucasian patients with NSCLC also showed more tumors from smokers expressed PD-L1 ≥ 50% than tumors from never smokers [15]. In addition, among our NSCLC patients who were smokers, a significantly higher proportion of heavy smokers than non-heavy smokers had high PD-L1 expression. This finding was mainly attributed to our patients who were smokers with adenocarcinoma in whom a significantly higher proportion of heavy smokers (44.1%) compared to non-heavy smokers (8.3%) had high PD-L1 expression. A similar finding among our SCC patients was not observed because of their much smaller number. However, among our patients who were smokers, the level of PD-L1 expression was not affected by whether the smoker was a current smoker or a former smoker.

Among our male patients who were smokers, a significantly higher proportion of heavy smokers (37.5%) had high PD-L1 expression compared to light and moderate smokers (13.5%). The effect of smoking intensity on the presence or absence of *EGFR* mutation and the level of PD-1 expression could not be analysed in our female patients because only 5 of them were smokers, all of whom were heavy smokers.

A significant dose-dependent relationship between the quantity of smoking in terms of pack-year and tumour mutation burden (TMB) in advanced lung adenocarcinoma has been shown by a previous study [39]. This emphasizes the need to quantify smoking history as a continuous variable rather than just categorizing lung cancer patients as ever smokers or never smokers. Smoking can influence TMB level through the accumulation of somatic mutations caused by carcinogens in tobacco. High TMB levels are associated with increased PD-L1 expression on tumor and immune cells of advanced NSCLC [40]. This may provide the mechanistic explanation of high PD-L1 expression in NSCLC of heavy smokers. Among smoker patients with PD-L1 expression ≥ 50%, PD-1 inhibitor monotherapy was shown by a study to be associated with better response and longer progression-free survival in heavy smokers compared to never/light smokers [41].

Apart from cigarette smoke, motor vehicle exhaust and ambient air pollution could upregulate PD-L1 expression in lung cancer patients [42]. As all our patients were living in the Klang Valley, their exposure to environmental air pollution should have been generally similar with the exception of cigarette smoking and smoking intensity. After adjusting for gender and smoking status, heavy smoking and *EGFR* wild-type tumors remained significantly associated with high PD-L1 expression in our patients with NSCLC and specifically also in those with adenocarcinoma.

There was no significant association between the level of PD-L1 expression and histologic subtypes of NSCLC in our study. Other studies have reported higher PD-L1 expression in adenocarcinoma compared to squamous cell carcinoma (SCC) [15, 36, 39] higher PD-L1 expression in SCC than in adenocarcinoma [40, 41] or no difference between the two histologic subtypes [42, 43]. The lack of association of PD-L1 expression with histology of our NSCLC patients could be due to the small number of SCC patients relative to the number of adenocarcinoma patients and about a fifth of our SCC patients were never smokers—more than half of whom had *EGFR* mutation.

## Conclusions

In conclusion, we have shown in this cohort of NSCLC patients more than half of whom were never smokers, high PD-L1 expression was more common in smokers than in never smokers, in *EGFR* wild-type than *EGFR*-mutant NSCLC and in heavy smokers among the smokers. On multivariate analysis, after adjusting for gender and smoking status, heavy smoking and *EGFR* wild-type tumors remained significantly associated with high PD-L1 expression in NSCLC and also in adenocarcinoma.

### Limitations of the study

The limitations of our study relate to it being a retrospective single center study with the attendant possibility of patient selection bias and potentially incomplete or inaccurate documentation. The age range of the study patients (32–89 years) was wide because this is a real-world study but the median age of the patients was 67 (interquartile range, 59–73) years. The other limitation was the test for driver mutations was limited to three most common ones, i.e. *EGFR* mutation, *ALK* and *ROS1* rearrangement. Since we did not use next-generation sequencing for molecular profiling, the number of tumors with these common driver alterations could have been higher and particularly some cases of *EGFR* exon 20 insertion mutations could have been missed. Only one patient was tested positive for *ROS1*. The very small number of patients who were positive for *ALK* did not allow any significant relationship to be established between *ALK* and PD-L1 expression. SCC was underrepresented in our study which limits relationship of PD-L1 expression with smoking to be analysed in this histologic subtype.

## Data Availability

The datasets used and/or analysed during the current study are available from the corresponding author on reasonable request.

## Abbreviations

*ALK*: Anaplastic lymphoma kinase
AJCC: American Joint Committee on Cancer
CECT: Contrast enhanced computed tomography
*EGFR*: Epidermal growth factor receptor
ESMO: European Society of Medical Oncology
FISH: Fluorescence in-situ hybridization
FFPE: Formalin-fixed paraffin embedded
ICI: Immune checkpoint inhibitor
IHC: Immunohistochemistry
MRI: Magnetic resonance imaging
NCCN: National Comprehensive Cancer Network
NGS: Next-generation sequencing
NSCLC: Non-small cell lung cancer
PD-L1: Programmed death-ligand 1
PET: Positron emission tomography
*ROS1*: C-ros oncogene 1
SCC: Squamous cell carcinoma
TKI: Tyrosine kinase inhibitor
TMB: Tumor mutation burden
TNM: Tumor, node and metastasis
TPS: Tumor proportion score
SPSS: Statistical Package for Social Sciences
